# Antioxidant and anti-inflammatory activity of Ocimum labiatum extract and isolated labdane diterpenoid

**DOI:** 10.1186/s12950-015-0049-4

**Published:** 2015-01-20

**Authors:** Petrina Kapewangolo, Justin J Omolo, Ronel Bruwer, Pascaline Fonteh, Debra Meyer

**Affiliations:** Department of Biochemistry, Faculty of Natural and Agricultural Sciences, University of Pretoria, Hatfield Campus, Pretoria, 0002 South Africa; Molecular Sciences Institute, School of Chemistry, University of the Witwatersrand, P.O. Box 2050, Johannesburg, South Africa; Present address: Chemistry & Biochemistry department, Faculty of Science, University of Namibia, Windhoek, 9000 Namibia; Present address: Department of Traditional Medicine, National Institute for Medical Research, P. O Box 9653, Dar es Salaam, Tanzania; Present address: Department of Biochemistry, Faculty of Sciences, University of Johannesburg, P.O. Box 524, Auckland Park, Johannesburg, 2006 South Africa

**Keywords:** *Ocimum labiatum*, Labdane diterpenoid, Inflammatory cytokines, Nitric oxide, Antioxidant, AP-1

## Abstract

**Background:**

Plants from the genus *Ocimum* are used as folk medicine for treating various diseases including inflammatory and immune-related diseases. Numerous reports have suggested plant extracts and their constituents as possible anti-inflammatory agents. Here, *in vitro* evidence of *Ocimum labiatum*’s immune-enhancing and antioxidant properties is presented for the first time.

**Methods:**

The anti-inflammatory effect of *O. labiatum* ethanolic extract and an isolated diterpenoid was determined using a cytometric bead array (CBA) technique. The effect on phytohemagglutinin (PHA)-induced nitric oxide (NO) production in peripheral blood mononuclear cells (PBMCs) was also assessed. A battery of antioxidant assays were used for detecting antioxidant activity while the anti-inflammatory mechanism was evaluated using an ELISA-based activator protein (AP-1) (c-Jun) assay. Cytotoxicity was determined on TZM-bl and PBMCs using a tetrazolium dye and confirmed by a novel label-free real-time assay.

**Results:**

A 25 μg/mL non-cytotoxic concentration of *O. labiatum* extract significantly (p < 0.05) inhibited the production of pro-inflammatory cytokines; IL-2, IL-4, IL-6 and IL-17A. Except for the dual acting pro- or anti-inflammatory cytokine, IL-6, which was upregulated, a non-cytotoxic 50 μM concentration of the isolated labdane diterpenoid compound significantly (p < 0.05) decreased the production of all the pro-inflammatory cytokines. In the anti-inflammatory pathway studies, the compound also inhibited AP-1 significantly (p < 0.05) at 50 μM. The extract demonstrated strong, dose dependent antioxidant activity with IC_50_ values ranging from 13 ± 0.8 to 54.86 ± 1.28 μg/mL while the terpene had no antioxidant property. The extract and diterpenoid decreased the production of the inflammatory mediator NO, at non-cytotoxic concentrations. The CC_50_ of the extract in TZM-bl and PBMCs was 62.6 ± 0.6 and 30.1 ± 0.4 μg/mL while that of the compound was 112.6 ± 0.2 and 70 ± 0.4 μM respectively. The real time studies confirmed tetrazolium dye assessed viability and also detected a unique growth pattern for the plant materials compared to untreated cells.

**Conclusions:**

*O. labiatum* extract demonstrated promising anti-inflammatory and antioxidant properties while the terpenoid showed anti-inflammatory but no antioxidant activity. The anti-inflammatory mechanism of the terpene was a result of inhibition of AP-1. These data represents promising first steps towards the development of naturally derived anti-inflammation drugs.

## Background

The majority (60%) of approved drugs are either directly isolated or derived from natural products [1]. *Ocimum labiatum*, commonly known as ‘pink sage’, belongs to the Lamiaceae family and is widely distributed in Southern Africa [2]. The plant is indigenously used for medicinal purposes, mostly antimicrobial [2,3]. Plants from the *Ocimum* genus are used traditionally to alleviate various disease symptoms such as pain, fever and inflammation, and the pharmacological activities of some extracts of these plants have been studied *in vitro* or *in vivo* without identifying the bioactive components [4-7].

Lamiaceae plants are generally rich in terpenoids; a diverse class of naturally occurring organic-chemicals derived from five-carbon isoprene units. Plant isolates containing terpenoids have been found to suppress nuclear factor kappa B (NF-κβ) signalling [8], a protein complex linked to the pathogenesis of inflammatory diseases, cancer, viral infection and autoimmune diseases [8]. In another study, semisynthetic labdane diterpenoid derivatives reportedly suppressed NF-κβ and nitric oxide (NO) production in macrophages [9]. NO is an inflammatory molecule produced by inducible NO synthase in macrophages and plays a role in immunoregulation [10,11]. Inhibitors of NO might be of therapeutic importance in preventing pathological conditions catalysed by inflammation [9-11]. Investigations of labdane diterpenoids suggest these compounds to have potential as alternative treatment for inflammatory diseases and further investigation is needed to identify the exact mechanism of action and pathways that are modulated by these compounds. Two labdane diterpenoid compounds were isolated from *O. labiatum* by Hussein et al. (2007) one of which inhibited *Mycobacterium tuberculosis* and the other demonstrated moderate anti-cancer properties both *in vitro* [12].

Plants from the Lamiaceae family are considered to be good sources of antioxidants due to the presence of high concentrations of phenolic compounds [13,14]. Antioxidants have the ability to dismutate reactive oxygen species (ROS) which are produced by the oxidation processes in various cells. Oxidative stress, caused by the accumulation of ROS in animal tissues, is a major cause of cell damage or death and is considered an instrumental process that leads to various cancers and other diseases [15]. In addition, ROS in low concentrations act as significant cell signalling molecules and regulates the biological conditions of cytokines, hormones and growth factors. High levels of free radicals, however, overcome the normal cellular antioxidant defences and end up being cytotoxic to the biological system [16]. These cumulative ROS are associated with a number of diseases including chronic inflammatory diseases [17-19]. ROS have also been reported to be involved in the activation of NF-κβ by pro-inflammatory cytokines such as Tumour necrosis factor (TNF)-α [16]. Given the importance of activated NF-κβ in inflammatory disease progression, suppression of this protein directly or through inhibition of ROS or pro-inflammatory cytokines preferably by antioxidants, remain therapeutically important because of the ability of the latter to combat pathogenic chain reactions initiated by free radicals.

The activator protein 1 (AP-1) is another transcription factor which regulates inflammatory cytokines and thus is being targeted as a way of circumventing inflammation [20]. AP-1 consist of dimeric transcription factors namely Jun, Fos and ATF subunits [21]. Inhibition of the c-Jun component of AP-1 results in the prevention of transcription of inflammatory genes and hence inflammatory cytokines since AP-1 is prevented from binding to transcription factors in the nucleus [22].

The onset of common human diseases such as autoimmunity and chronic infections is characterized by a dysregulation of the T helper cell type 1 (Th1) and Th2 cytokine balance [23]. Interleukin (IL)-6 has been reported to be responsible for Th17 cells induction to secrete IL-17, a pro-inflammatory cytokine [24]. Since cytokines are central mediators in major inflammatory diseases and impact one another’s production and action [23], it is important to simultaneously measure more than one cytokine from all 3 subsets; Th1/Th2/Th17. This will allow for better assessment of immune and inflammation status with these proteins as potential indicators in prognostic and drug discovery studies [23,25].

Although many species of the *Ocimum* genus have been extensively investigated *in vitro*, this is not the case with *O. labiatum*. In this study, *O. labiatum* was investigated for anti-inflammatory properties through inhibition or suppression of pro-inflammatory cytokines using the human Th1/Th2/Th17 cytometric bead array assay which allows for the quantification of multiple cytokines in a single sample. The effect of *O. labiatum* on NO production was also evaluated because there is on-going research for potential anti-inflammatory agents from nature due to adverse side effects and high costs of existing anti-inflammatory drugs [26]. The antioxidant potential of *O. labiatum* as well as the anti-inflammatory pathway was also investigated.

## Methods

### Plant extraction and compound isolation

Fresh leaves (894.6 g) of *O. labiatum* were collected during February (2012) from the Botanical garden of the University of Pretoria. Plant identification was done in the H.G.W.J Schweikerdt herbarium of the University and a voucher specimen (117693) is kept in the herbarium.

Fresh leaves were blended in ethanol and filtered. The filtrate was evaporated under reduced pressure at 50°C using a rotary evaporator (Buchi, Flawil, Switzerland) and the residue was dissolved in ethyl acetate to obtain a lipophilic fraction. The ethyl acetate fraction was transferred to a pre-weighed vial and was evaporated to dryness at room temperature. The dried extract was stored in the dark at 4°C until use. The extract was weighed out as needed and reconstituted in dimethyl sulfoxide (DMSO) before each biological assay. Reconstituting in DMSO surface sterilized the extract. Further dilutions to the desired extract concentrations were done in either cell culture media or buffer depending on the type of biological assay performed.

The ethyl acetate fraction (31.1 g) was subjected to column chromatography (Si gel 70–230 mesh) eluting with hexane (1.5 L) then with hexane-ethyl acetate (9:1, 4:1, 7:3, 3:2, 1:1, 3:7; 1.5 L each) and finally with ethyl acetate (1.5 L), collecting fractions of 500 mL each. The last fraction, with 100% ethyl acetate, yielded a pure compound in the form of white crystals (65 mg).

**Figure 1:**
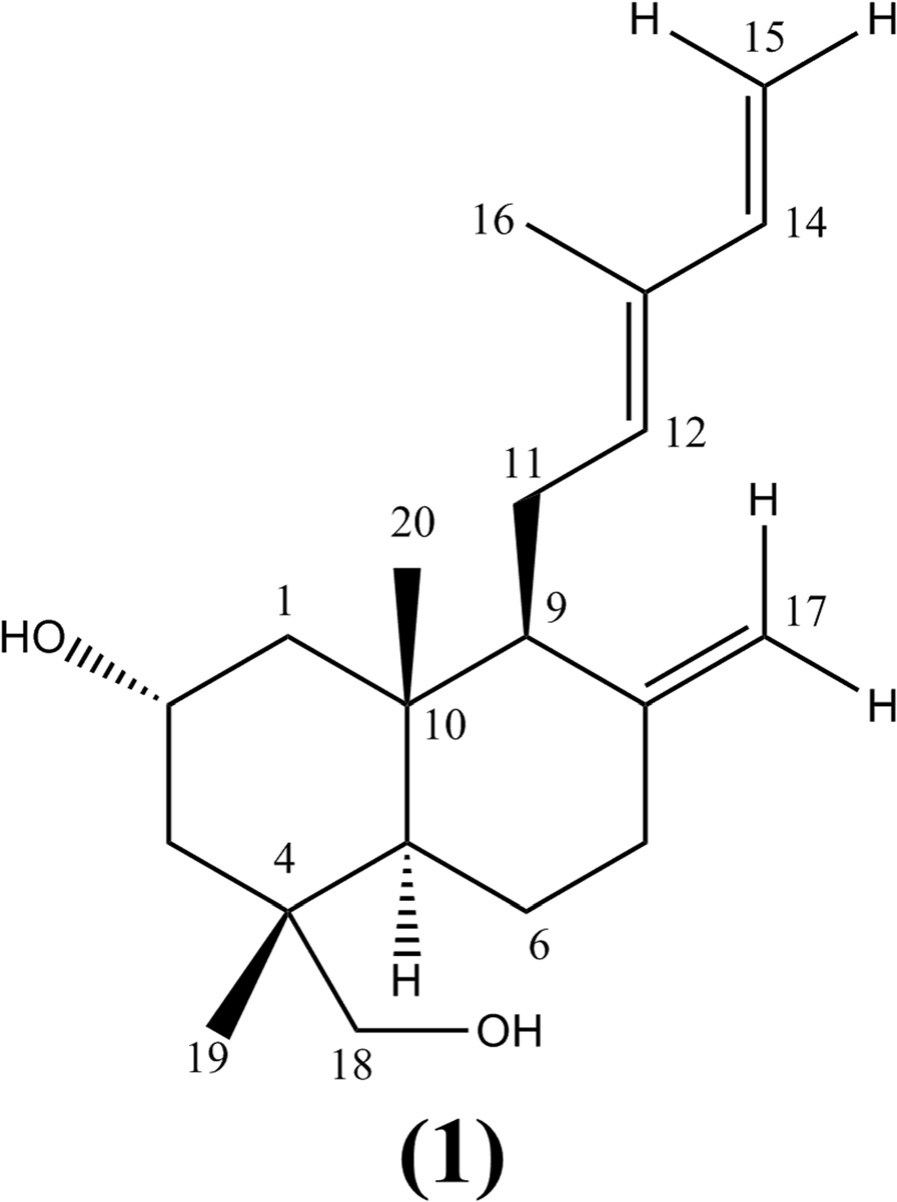
**Chemical structure of the labdane diterpenoid Labda-8(17), 12** ***E*** **,14-triene-2R,18-diol.**

Labda-8(17),12*E*,14-triene-2R,18-diol (Figure 1): White crystals (C_20_H_32_O_2_); ^1^H NMR (CDCl_3_, 500 MHz): δ 6.32 (1H, dd, J = 10.5, 17.5 Hz, H-14), 5.40 (1H, t, J = 6.0, 12.5 Hz, H-12), 5.05 (1H, d, J = 17.5 Hz, H-15a), 4.88 (1H, d, J = 11.0 Hz, H-15b), 4.86 (1H, d, J = 1.5 Hz, H-17a), 4.49 (1H, d, J = 1.0 Hz, H-17b), 3.97 (1H, m, H-2), 3.42 (1H, d, J = 11.0 Hz, H-18a), 3.18 (1H, d, J = 11 Hz, H-18b), 2.38 (1H, m, H-11a), 2.37 (1H, m, H-11b), 2.03 (2H, m, H-7), 1.88 (1H, d, J = 10.5 Hz), 1.75 (3H, s, H-16), 1.63 (2H, m, H-6), 1.47 (1H, dd, J = 2.5, 12.5 Hz, H-5), 1.41 (2H, d, J = 11.5 Hz, H-3), 1.05 (2H, t, J = 11.5, 23.0 Hz, H-1), 0.81 (3H, s, H-20), 0.80 (3H, s, H-19); ^13^C NMR (CDCl_3_, 125 MHz): δ 147.4 (C, C-8), 141.5 (CH, C-14), 133.6 (C, C-13), 133.4 (CH, C-12), 110.0 (CH_2_, C-15), 108.5 (CH_2_, C-17), 71.5 (CH_2_OH, C-18), 65.5 (CHOH, C-2), 56.8 (CH, C-9), 47.9 (CH_2_, C-1), 47.5 (CH, C-5), 44.6 (CH_2_, C-3), 40.7 (C, C-10), 39.4 (C, C-4), 37.5 (CH_2_, C-7), 23.5 (CH_2_, C-6), 23.3 (CH_2_, C-11), 18.5 (CH_3_, C-19), 15.8 (CH_3_, C-20), 11.8 (CH_3_, C-16); MS *m/z* 304.2402 (calculated for C_20_H_32_O_2_ [M^+^] 304.2402).

The ^1^H and ^13^C NMR spectra of compound **1** displayed resonances for an exocyclic methylene group H-17 at δ 4.86 (1H, d, 1.5) and 4.49 (1H, d, 4.49) both attached to carbon at δ 108.5. The spectra further showed the presence of C-9 side chain which was identical to those reported data by Hussein *et al*., 2007. The ^1^H NMR spectra showed the presence of methine proton in a cyclohexane ring at δ 3.97 (1H, m), placed between two methylene substituents at δ 1.05 (2H, t, 11.5, 23.0) and 1.41 (2H, d, 11.5), suggesting C-2 was attached to a hydroxyl substituent, possibly as part of the labdane hydrocarbon skeleton. The spectroscopic data showed an AB system at δ 3.42 and 3.18 (1H, d, J = 11.0 each) of hydroxymethylene group attached to quaternary carbon C-4 (δ 71.5). The position of the hydroxyl group at the C-18 (δ 71.5) was further confirmed by the HMBC spectrum which showed correlation between the hydoxymethylene protons and the C-3 (δ 44.6), C-4 (δ 39.4), C-5 (δ 47.5) and C-19 (δ 18.5).

The ^13^C NMR and DEPT spectra showed the presence of twenty carbon signals. These included, four quaternary carbons C-4, C-8, C-10 and C-13 at δ 39.4, 147.4, 40.7 and 133.6 respectively; one oxymethine carbon C-2 at δ 65.5; four methine carbons C-5, C-9, C-12 and C-14 at δ 47.5, 56.8, 133.4 and 141.5 respectively; one oxymethylene carbon C-18 at δ 71.5; seven methylene carbons C-1, C-3, C-6, C-7, C-11, C-15 and C-17 at δ 47.9, 44.6, 23.5, 37.5, 23.2,110.0 and 108.5 respectively; and three methyl carbons C-16, C-19 and C-20 at δ 11.8, 18.5 and 15.8 respectively as shown in Table 1.

**Table 1:** **NMR spectral assignments for compound 1**

**Carbon number**	**δ**_**C**_	**δ**_**H**_ **, J (Hz)**	**HMBC**	**COSY**
C-1 (CH_2_)	47.9	1.05 (2H, t, 11.5, 23.0)		
C-2 (CHOH)	65.5	3.97 (1H, m)		
C-3 (CH_2_)	44.6	1.41 (2H, d, 11.5)		
C-4 (C)	39.4			
C-5 (CH)	47.5	1.47 (1H, dd, 2.5, 12.5)		
C-6 (CH_2_)	23.5	1.63 (2H, m)		
C-7 (CH_2_)	37.5	2.03 (2H, m)		
C-8 (C)	147.4			
C-9 (CH)	56.8	1.88 (1H, d, 10.5)		
C-10 (C)	40.7			
C-11 (CH_2_)	23.2	2.38 (1H, m)		H-11b
		2.37 (1H, m)		H-11a
C-12 (CH)	133.4	5.40 (1H, t, 6.0, 12.5)	C-9, C-11, C-14, C-16	
C-13 (C)	133.6			
C-14 (CH)	141.5	6.32 (1H, dd, 10.5, 17.5)	C-13, C-16	
C-15 (CH_2_)	110.0	5.05 (1H, d, 17.5)	C-13, C-14	H-14
		4.88 (1H, d, 11.0)		H-14
C-16 (CH_3_)	11.8	1.75 (3H, s)		
C-17 (CH_2_)	108.5	4.86 (1H, d, 1.5)	C-7, C-9	H-17b
		4.49 (1H, d, 1.0)		H-17a
C-18 (CH_2_OH)	71.5	3.42 (1H, d, 11.0)	C-3, C-4, C-5, C-19,	H-18b
		3.18 (1H, d, 11.0)		H-18a
C-19 (CH_3_)	18.5	0.80 (3H, s)		
C-20 (CH_3_)	15.8	0.81 (3H, s)		

The COSY spectrum depicted mostly correlations of the adjacent protons H-11a,b; H-14a,b; H-17a,b and H-18a,b. From the NMR spectroscopic data the molecular formula of compound **1** was determined to be C_20_H_32_O_2_, corresponding to a theoretical exact mass of 304.24. The MS data showed a molecular ion M^+^ peak at *m/z* 304.24 confirming the structure of compound **1**. The ^1^H and ^13^C NMR spectroscopic data for compound **1** compared well with literature data [12].

### Viability of TZM-bl cells using MTT

The effect of *O. labiatum* extract and isolated compound on the viability of TZM-bl cells was determined by quantifying the amount of a formazan product metabolized by viable cells from 3-(4,5-dimethylthiazol-2-yl)-2,5-diphenyltetrazolium bromide (MTT) solution (Sigma, MO, USA) as previously reported [27]. TZM-bl cells were maintained in complete Dulbecco’s Modified Eagle Medium (DMEM; Sigma, MO, USA); containing antibiotics and foetal bovine serum. Cells were plated in 96 well plates (Corning Incorporated, Corning, USA) at 1 × 10^4^ cells per well and were treated with crude extract at final concentrations of 100, 50, 25, 12.5, 6.25 and 3.125 μg/mL. In the case of the compound, the final concentrations were 3.125-100 μM. Viability was determined after 72 h incubation (37°C, 90% humidity, 5% CO_2)_. Control wells included a negative control (cells and medium only), blank control for the extract (extract and medium only), a toxicity control auranofin; a known toxic compound with antitumour activity [28], and a DMSO control (percentage of DMSO similar to extracts in cells to ensure that this solvent did not cause cell death). The plates were read at 550 nm using a microtiter plate reader (Multiskan Ascent; Thermo Labsystems; MA, USA), a reference wavelength of 690 nm was used and the percentage viability was calculated relative to untreated control cells. Fifty percent cytotoxic concentration (CC_50_) of the extract and compound was obtained using Graphpad Prism (Graphpad Software Inc. CA, USA). This was computed as the concentration of the extract/compound that reduced cell viability by 50% when compared to controls.

### Viability of PBMCs using MTS

Ethical approval for obtaining blood samples from consenting donors was granted by the Faculties of Natural and Agricultural Sciences and Health Sciences Ethics Committees (EC080506-019; 163/2008, University of Pretoria, South Africa). Freshly isolated healthy peripheral blood mononuclear cells (PBMCs) were suspended in complete RPMI 1640 (Sigma, MO, USA) medium (containing antibiotics and foetal bovine serum). Cells were then stimulated with phytohemagglutinin-protein (PHA-P, 4 μg/mL), plated in 96 well plates (Corning Incorporated, Corning, USA) at 1 × 10^5^ cells per well and treated with the extract and compound at final concentrations of 100, 50, 25, 12.5, 6.25 and 3.125 μg/mL. The number of viable cells was detected after 72 h using 3-(4,5-dimethylthiazol-2-yl)-5-(3-carboxymethoxyphenyl)-2-(4-sulfophenyl)-2H-tetrazolium, inner salt (MTS) solution (Promega Corporation, WI, USA). Control wells included a toxicity control auranofin and the plates were read at 492 nm (reference wavelength of 690 nm). The percentage viability was calculated relative to an untreated control of cells only and the CC_50_ values were determined using Graphpad Prism (Graphpad Software Inc. CA, USA).

### Real time cell analysis

Confirmatory cytotoxicity analysis for the crude extract and isolated compound was performed using a real time cell electronic sensing (RT-CES) device, xCELLigence (Roche Diagnostics, MA, Germany) to monitor proliferation of the TZM-bl cells in the presence of the extract and isolated compound. A detailed procedure was followed as previously described [29]. The xCELLigence system monitors cellular events in real time without the incorporation of labels by measuring electrical impedance across interdigitated gold micro-electrodes integrated on the bottom of special tissue culture plates. Increasing attachment of cells to the electrodes increases electrode impedance which is displayed as cell index (CI) [30,31]. Cell titration was carried out as recommended by the manufacturer in order to determine an optimal cell number that reaches an index of ±1 after 24 h, before treatment with the samples. Each well was seeded with 10,000 cells, which was the ideal cell number obtained from the titration. The CC_50_ of *O. labiatum* extract obtained with an MTT assay was tested alongside 10 μM auranofin as a positive control for toxicity. Untreated cells were also included as controls. Cells were first allowed to adhere for 24 h before treating them with extracts. The cellular effects of the extracts were monitored for 72 h and CI values were recorded. The compound was applied in the same manner. One concentration of the compound, 112.6 μM, CC_50_ determined with MTT was also monitored for its effect in real time for 72 h.

### Antioxidant assays

The antioxidant potential of the extract and terpene were investigated using four different antioxidant assays since antioxidant mechanisms could include different reactions with radical species. For these assays, all reagents were obtained from Sigma Aldrich (Missouri USA) unless stated otherwise. Ascorbic acid was used as the positive control for all these assays at concentrations ranging from (0.078 μg/mL to 100 μg/mL).

#### DPPH antioxidant assay

The potential antioxidant activity of *O. labiatum* extract and Labda-8(17),12*E*,14-triene-2R,18-diol was assessed on the basis of the scavenging ability of the test samples towards a stable 2,2-diphenyl-1-picrylhydrazyl (DPPH) free radical. Test samples were prepared at a starting concentration of 100 μg/mL after mixing with 90 μM DPPH ethanol solution. A series of concentrations were tested for the samples in order to determine the 50% inhibitory concentration (IC_50_). Samples were incubated with DPPH in the dark for 30 min at room temperature. Absorbance values were measured at 550 nm (Multiskan Ascent; Thermo Labsystems; MA, USA) and converted into percentage of antioxidant activity [32]. Background control was samples in ethanol only and a DPPH only control was also include. IC_50_ was determined using Graphpad Prism (Graphpad Software Inc. CA, USA). The percentage inhibition was calculated by using the formula:

$$ 100\ \mathit{\hbox{--}}\ \left[\left( Sample\  Optical\  density\ (OD)\ \mathit{\hbox{--}}\  Sample\  background\ OD\right)/\left( DPPH\  only\ OD\right) \times 100\right] $$

#### Ferric reducing antioxidant power (FRAP)

The FRAP assay was performed a previously described, with minor modifications [33,34]. The terpenoid compound and extract (0.781 to 100 μg/mL) was dissolved in DMSO (5%) and further diluted with double distilled deionized water.

Freshly prepared FRAP reagent (20 mL acetate buffer, 2 mL TPTZ solution, 2 mL ferric chloride solution and 2.4 mL autoclaved dH_2_O) was heated to 37°C. A reagent blank, ascorbic acid calibrations (0.078 μg/mL to 10 μg/mL) and samples were submitted to the FRAP assay as follows: 8 μl of sample and 240 μl FRAP reagent were added to 96 well plates, mixed and incubated for 10 minute. Absorbance readings were obtained at 595 nm and plotted as *(Sample Optical density (OD) – Sample background OD-blank)* against compound or extract concentration.

#### Cupric reducing antioxidant capacity (CUPRAC)

This assay was performed as previously described [35]. The terpenoid compounds and extract concentrations ranged from 0.781 μg/mL to 100 μg/mL and that for ascorbic acid ranged from 0.078 μg/mL to 10 μg/mL. The sample (50 μl) and 150 μl CUPRAC reagent consisting of 10 mM Copper(II)chloride, 7.5 mM neucoproine (AEC Amersham) and ammonium acetate buffer (1% (v/v) acetic acid, 0.1 M ammonium acetate, pH7) were mixed and incubated for 30 minutes at room temperature before absorbance readings were obtained at 450 nm. An experiment blank and sample background controls were also included. The data was plotted as *(Sample Optical density (OD) – Sample background OD-blank)* against compound or extract concentration.

#### Crocin bleaching assay using 2,2′-azobis (2-amidonopropane) hydrochloride (AAPH)

*Reagents*: PBS (27 mM KCl, 18 mM KH_2_PO_4_, 1.4 M NaCl, 0.1 M Na_2_HPO_4_ and autoclaved dH_2_O), 20 μM crocin solution (crocin dissolved in 30% methanol), AAPH solution (5 mg/mL AAPH dissolved in 1x PBS).

This assay was performed according to a previously described method [36]. Fifty microliters of the terpenoid compound and extract (0.78 μg/mL to 100 μg/mL) and ascorbic acid (0.078 μg/mL to 10 μg/mL) were added to 100 μl crocin solution and 100 μl of AAPH solution. These were mixed and incubated for 1 hour at room temperature before absorbance readings were obtained at 450 nm. The absorbance readings were plotted as *(Sample Optical density (OD) – Sample background OD-blank)* against compound or extract concentration.

### Cytokine quantitation using cytometric bead array (CBA)

Ethical approval for obtaining blood samples from consenting donors was granted by the Faculties of Natural and Agricultural Sciences and Health Sciences Ethics Committees (EC080506-019; 163/2008, University of Pretoria, South Africa). Sample preparation: Blood samples were taken from healthy volunteers; 9 individuals for extract and 14 for compound. Freshly isolated PBMCs stimulated with PHA-P (4 μg/mL), were seeded at 1 × 10^6^ cells/well in order to get enough cytokine produced for quantitative detection. Incubation of the cells with non-cytotoxic concentrations of the extract (25 μg/mL) and compound (50 μM) was done for 24 h. The supernatant was collected and stored at −20°C until testing.

Quantitation: Cytokine levels were analysed in the tissue culture supernatant using a BD CBA human Th1/Th2/Th17 cytokine kit (BD Biosciences, CA, USA). The CBA kit simultaneously measured IL-2, IL-4, IL-6, IL-10, TNF, interferon gamma (INF-ɣ) and IL-17A protein levels in a single sample using a FACSArray Bioanalyzer (BD Biosciences, CA, USA). The assay was performed according to the manufacturer’s instructions. Briefly, the supernatant was thawed and 50 μl of each sample was mixed with the cytokine capture beads and the detector reagent, phycoerythrin (PE)-conjugated detection antibodies, to form sandwich complexes. The intensity of PE fluorescence of each sandwich complex reveals the concentration of that cytokine [25]. The limits of detection for each cytokine was as follow: 2.6 pg/mL for IL-2, 4.9 pg/mL for IL-4, 2.4 pg/mL for IL-6, 4.5 pg/mL for IL-10, 3.8 pg/mL for TNF, 3.7 pg/mL for IFN-ɣ and 18.9 pg/mL for IL-17A.

### AP-1 (c-Jun) inhibition studies

The AP-1 group of transcription factors binds to the enhancer/promoter region of various cytokine genes in activated cells [37]. The extract and isolated compound were also evaluated to determine inhibition of the c-Jun component of AP-1 as an indication of prevention of transcription of inflammatory genes [22]. The Abcam c-Jun (Ps73) ELISA kit (Abcam®, Cambridge, UK) was used in the detection of PHA-P induced AP-1 from PBMCs and the assay performed according to the manufacturer’s protocol. PBMCs (1 × 10^6^ cells/mL) pretreated with or without the extract and diterpenoid compound were collected and washed with phosphate buffered saline. The cells were then lysed and antibody mix was added and incubated for 1 h in a microplate. The microplate was washed before the substrate was added for colour development. A stop solution was added to stop the reaction after 10 min and the fluorescence signal obtained at 544/590 nm using a Fluoroskan Ascent® plate reader (Labsystems, Helsinki, Finland).

### Nitrite and nitrate detection by colorimetric assay

The effect of extract and isolated compound on NO production was studied using an NO assay colorimetric kit (Calbiochem, CA, USA). In aqueous solution, NO is rapidly converted to nitrate and nitrite. Hence, for accurate determination of the total NO generated, both nitrate and nitrite levels must be monitored. Spectrophotometric quantitation of nitrite using only the Griess reagent does not measure nitrate. Therefore, the NADH-dependent enzyme nitrate reductase is used to convert the nitrate to nitrite prior to quantitation using the Griess reagent. NO was measured from PBMCs by plating cells in 96 well plates at 2.0 × 10^5^ cells/well. Cells were pre-incubated for 1 h with non-cytotoxic concentrations of the crude extract (25 μg/mL) and labdane diterpenoid (50, 25 and 10 μM) before stimulating for NO with a non-cytotoxic concentration of PHA-P, 25 μg/mL and further incubating for 24 h. After the 24 h incubation, cell culture supernatant (50 μl) was collected and incubated with 1 U/μl of nitrate reductase in the presence of 0.2 mM NADH and 50 mM MOPS buffer, pH 7.0. After 20 min, Griess reagent was added and further incubated for 5 min at room temperature. The colour was read at 550 nm (Multiskan Ascent; Thermo Labsystems; MA, USA). A standard curve was generated, using freshly prepared 0–100 μM potassium nitrate dissolved in assay buffer, to quantitate unknown nitrite in samples.

### Statistical analysis

Data for all experiments is presented as the mean ± standard deviation (n = 3-6). Since cytokine profiles vary from person to person, the cytokine concentrations obtained for each individual were log transformed in order to standardize the data and make it more comparable [25,38]. Significant differences were estimated using Graphpad Prism 5 (Graphpad Software Inc. CA, USA) and Student’s t test for unpaired observations. A p < 0.05 was considered significant.

## Results

### Cytotoxicity

The CC_50_ of crude *O. labiatum* extract was 62.6 ± 0.6 μg/mL for TZM-bl cells and 30.1 ± 0.4 μg/mL in PBMCs determined by viability dye MTT and MTS respectively. The PBMCs (1 × 10^5^ cells/well) used in this study did not adequately metabolize the viability dye MTT but properly metabolized MTS which is why the latter dye was used in the PBMC analyses and MTT for TZM-bl assessments. Tetrazolium dyes are metabolized differently by different cell types. Considerable evidence indicates that the reduction of MTS occurs at the cell surface or at the level of the plasma membrane via trans-plasma membrane electron transport and not inside a cell as is the case with MTT [39]. It is therefore possible that MTT was unable to properly penetrate the membrane of PBMCs for proper intracellular metabolism and MTS which is metabolized on the cell-surface was easily metabolized by these mononuclear cells.

**Figure 2:**
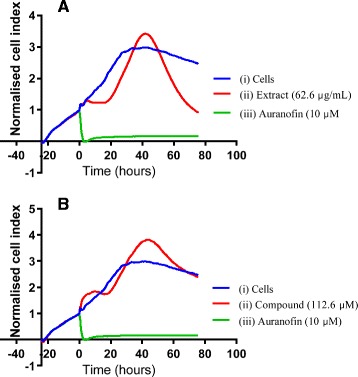
**Real time monitoring of** ***O. labiatum*** **effect on the viability of TZM-bl cells.** Cells were exposed to 62.6 μg/mL of extract **(A)** and 112.6 μM compound **(B)**, which were the CC_50_ values determined in a tetrazolium dye assay. Auranofin (iii) was used as a positive control for toxicity and caused cell death almost from the time of addition until end of incubation period (72 h). A unique growth pattern was observed in extract treated cells with uptake in the first 48 hours followed by extract content being metabolized by the cells resulting in an increase in cell index above that of untreated cells which following prolonged exposure is reduced to half cell index of control cells (i) at 72 h. RT-CES demonstrated low cytotoxicity for compound treated cells 72 h similar to that of untreated cells (i). For the compounds, uptake and metabolism was similar to that of extract however, elevated cell indices was maintained. Data was normalized against the time point before extract addition.

Cytotoxicity of the crude extract was confirmed with RT-CES. RT-CES monitored the effect of the extract on cell viability (Figure 2A), in real-time and confirmed the CC_50_ value obtained with MTT. At 72 h post treatment, the cell index for untreated cells (i) was 2.5. Cells treated with 62.6 ± 0.6 μg/mL crude extract (CC_50_) resulted in sample uptake (dip in cell growth before 24 h) and metabolisation (hump) as demonstrated by the pattern (ii) in Figure 2A. When the extract was properly metabolized, a reduction in cell viability was observed. About 50% (half of cell index value of control cells) of treated cells remained viable at 72 hours of incubation.

The cytotoxicity of the labdane diterpenoid compound, Labda-8(17),12*E*,14-triene-2R,18-diol, was also studied using viability dyes MTT and MTS and it was found to cause CC_50_s of 112.6 (34.3 μg/mL) ±0.2 and 70 (21.3 μg/mL) ±0.4 μM in TZM-bl and PBMCs respectively. The cytotoxicity results were again confirmed with RT-CES and these results are depicted in Figure 2B. Extract uptake was observed before 24 h and metabolization of the sample resulted in a cell index value of 2.5 (ii) equivalent to that of untreated control cells (i) at 72 h. RT-CES results indicated that the CC_50_ of the compound obtained in TZM-bl cells was not cytotoxic. Since RT-CES does not involve the use of dye uptake or invasive methods to measure cell status, this suggests this technique to be more sensitive and accurate than MTT.

### Antioxidant activity

**Figure 3:**
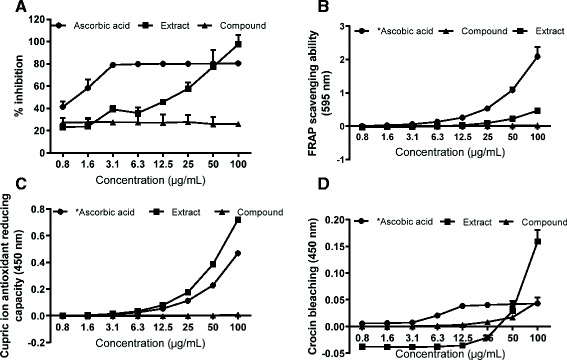
**Antioxidant effect of the** ***O. labiatum*** **extract (squares) and the isolated compound (triangles).** The DPPH free radical scavenging, the FRAP, cupric ion reducing activity, and crocin bleaching effect are shown in **A**, **B**, **C** and **D** respectively (n = 5-6.). The DPPH data is shown as percentage inhibition. The IC_50s_ for the extract were 13 ± 0.8 μg/mL, 53.62 ± 0.57, 47.32 ± 0.76 μg/mL and 54.86 ± 1.28 μg/mL respectively. Ascorbic acid was used as positive control. The extract exhibited strong antioxidant activity while the diterpenoid compound did not. *tested in two fold serial dilutions from 0.08-10 μg/mL.

The antioxidant effect of the test samples and ascorbic acid obtained in the DPPH, FRAP, CUPRAC and crocin bleaching antioxidant assays are shown in Figure 3. For all the assays, a dose dependent antioxidant activity was observed for the extract with IC_50_ values of 13 ± 0.8 μg/mL, 53.62 ± 0.57, 47.32 ± 0.76 μg/mL and 54.86 ± 1.28 μg/mL respectively. The IC_50_ of ascorbic acid for the individual assays was found to be 1.1 ± 0.03, 4.74 ± 0.60, 5.29 ± 0.03 and 6.69 ± 0.004 μg/mL respectively. For all the assays, the labdane diterpenoid compound demonstrated minimal to no antioxidant activity (<50%) for all tested concentrations. Each assay was performed at least 5 times.

### Effect of *O. labiatum* on cytokine production

**Figure 4:**
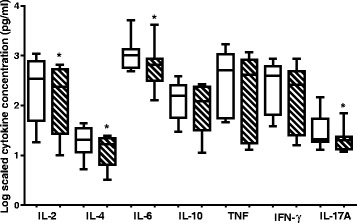
**Effects of a non-toxic concentration of** ***O. labiatum*** **extract (25 μg/mL) on cytokine production in PBMCs.** Cytokines were quantified using the CBA kit and flow cytometry. Cytokine concentrations were log transformed in order to make the data comparable. Control treatments are represented by unshaded bars whereas shaded bars represent extract treated samples. The crude extract significantly (*p < 0.05) down-regulated levels of IL-2, IL-4, IL-6 and IL-17A.

Using the Cytometric Bead Array (CBA) human Th1/Th2/Th17 cytokine kit (BD Biosciences, CA, USA), 7 cytokines, IL-2, IL-4, IL-6, IL-10, TNF, INF-ɣ and IL-17A, were analysed and quantified (Figure 4) following exposure of PBMCs to plant material. *O. labiatum* crude extract was tested for anti-inflammatory properties at 25 μg/mL, a non-cytotoxic concentration. Cytokine production in untreated PBMCs was used as a control for comparison to the production of cytokines in extract treated PBMCs. The extract significantly (p < 0.05) decreased the production of pro-inflammatory cytokines IL-2, IL-4, IL-6 and IL-17A (Figure 4). For IL-10, INF-ɣ and TNF, there was no significant (p > 0.05) difference detected in untreated and extracted treated samples even though production of those cytokines was lowered by the extract.

### Effect of labda-8(17),12*E*,14-triene-2R,18-diol on pro-inflammatory cytokines

**Figure 5:**
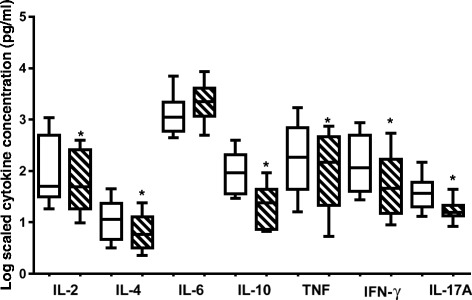
**Influence of labda-8(17),12** ***E*** **,14-triene-2R,18-diol on cytokine production in PBMCs as quantitated by CBA analysis and flow cytometry.** Cells were treated with a non-toxic concentration of 50 μM. Concentrations obtained of tested cytokines were log transformed in order to make the data comparable. Control treatments are unshaded whereas compound treated cells are shaded. IL-6 was the only cytokine that was up-regulated by the compound. The rest of the cytokines were significantly (*p < 0.05) down-regulated.

The anti-inflammatory property of the purified labdane diterpenoid was also studied using the CBA human Th1/Th2/Th17 cytokine kit (BD Biosciences, CA, USA). The compound was tested at 50 μM, a non-toxic concentration. The results obtained were compared to untreated cells. The compound was found to significantly (p < 0.05) inhibit the production of all cytokines tested except for IL-6 as illustrated in Figure 5. The production of IL-6, which acts as both an anti- and pro-inflammatory cytokine, was significantly (p < 0.05) up-regulated.

### AP-1 inhibition by the labdane diterpenoid

**Figure 6:**
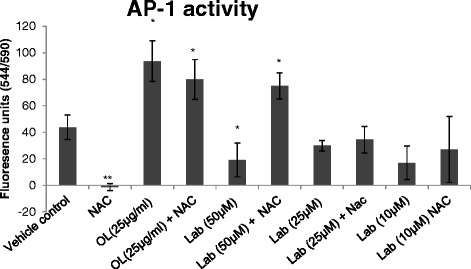
**The effect of the isolated compound and plant extract on AP-1.** The isolated labdane diterpenoid (Lab) and the extract (OL) were tested for activity against the c-Jun component of AP-1 (n = 3) in PHA stimulated PBMCs. Lab significantly (p < 0.05) inhibited AP-1 in PBMCs at 50 μM. NAC (100 μg/mL) was used as positive control and significantly inhibited AP-1 (a). Co-treatment with NAC resulted in stimulation of AP-1 production. At 25 μg/mL, the plant extract significantly stimulated AP-1 in PBMCs (p < 0.05).

The effect of the terpenoid (50 μM, 25 μM & 10 μM) and the extract (25 μg/mL & 10 μg/mL) from the plant, *O. labiatum* were tested for activity against c-Jun component of AP-1 (n = 3) in PHA stimulated PBMCs. The isolated compound significantly inhibited (p < 0.05) AP-1 at 50 μM, but not at lower concentrations (Figure 6). N-acetyl cysteine (NAC) which was used as the positive control significantly inhibited AP-1 (p < 0.005). When cells were co-treated with the terpenoid compound and NAC, AP-1 was significantly stimulated (p < 0.05) at 50 μM, but not at lower concentrations suggesting that the anti-inflammatory activity of the terpenoid was as a result of inhibition of the AP-1 pathway. Cells treated with extract alone, as well as co-treated with extract and NAC significantly (p < 0.05) stimulated AP-1 production (Figure 6).

The effect of the compound on NF-kB from nuclear extracts obtained from PBMCs was assessed using the NF-kB (p65) transcription factor assay kit (Rockland Immunochemicals, Gilbertsville, PA, USA). The compound had no appreciable effect on NF-kB at tested concentrations (data not shown).

### NO levels in culture supernatant reduced by *O. labiatum* and labdane diterpenoid

**Figure 7:**
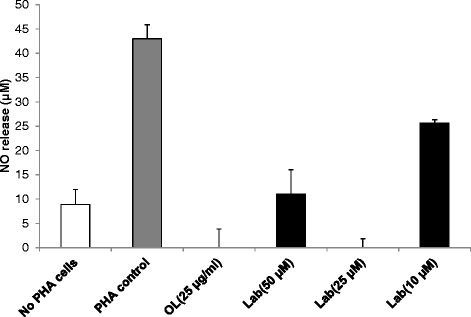
**Inhibition of NO release by** ***O. labiatum*** **extract and labdane diterpenoid.** PBMCs were pre-incubated for 1 h with the indicated concentrations of the extract (OL) and compound (Lab) and then stimulated with 25 μg/mL PHA for 24 h. Untreated cells were used as a control. Nitrite accumulation was measured with the Griess reagent. Extract and compound concentrations used were non-toxic. The results were expressed as mean ± SD.

The effects of the extract and compound on PHA-induced NO production in PBMCs were investigated by quantitating nitrite in culture medium using the Griess reaction. Unstimulated control cells, after 24 h of incubation, produced a negligible amount of NO (<10 μM). NO production in PHA stimulated cells was evident (43 μM), while no significant levels of NO were detected in the extract treated cells as illustrated in Figure 7. The three concentrations tested for the labdane diterpenoid compound significantly (p < 0.05) decreased the level of NO in the cells with 25 μM of the compound being more significant by reducing NO production to an undetectable level (Figure 7).

## Discussion

The data obtained in this study suggest that *O. labiatum* has immune supportive activities in addition to direct antioxidant abilities. RT-CES proved to be a more sensitive technique for detecting the effect of treatment on cell viability. The technique detected a unique cell growth pattern with extract and compound uptake and metabolism which would not have been observed with viability dyes. The use of conventional methods such as MTT or MTS has been reported to be insufficient if used alone since these methods only produce end-point data [40]. RT-CES, which is label-free, is able to capture important effects such as the onset and rate of cytotoxicity as well as other proliferation dynamics such as cytostasis [29]. Sensitivity and accurate reflection using RT-CES has been attributed to the ability of the technique to reflect on the cell concentration and changes in cell morphology more than viability dyes [40].

*O. labiatum* extract exhibited strong antioxidant activity while the isolated labdane diterpenoid, Labda-8(17),12*E*,14-triene-2R,18-diol, was not a good antioxidant suggesting the antioxidant ability of the crude extract to be due to the presence of other compound(s). Non-terpenoid compounds present in the extract such as flavonoids are more likely to be responsible for antioxidant activity of plant extracts. Flavonoids, vitamins and polyphenols present in plants are known to be powerful antioxidants [41].

The bead array technology used in this study allowed for simultaneous detection of multiple cytokines in a single sample. Because cytokines influence one another, an imbalance in especially Th1 and Th2 cytokines can lead to the pathogenesis of acute and chronic inflammatory diseases [23]. Therefore, in studies where cytokines are investigated as prognostic indicators [25], for therapeutic uses [42] or to assess their modulation by potential drugs [43], it is important to study an array of these molecules representative of all 3 subsets (Th1/Th2/Th17) as was done here.

Lamiaceae terpenoids have been documented to have anti-inflammatory as well as antitumor effects [44]. The isolation of the compound Labda-8(17),12*E*,14-triene-2R,18-diol from *O. labiatum* was first reported in 2007 [12]. The only investigations done on the labdane diterpenoid by these authors were anti-cancer testing against breast cancer cells and an anti-tuberculosis study in which the compound did not inhibit *M. tuberculosis* activity at the highest concentration tested but showed moderate cytotoxic effects against a breast cancer cell line [12]. According to the literature, some labdane diterpenoid compounds have been shown to possess immune enhancing properties [9] which encouraged the investigation of these specific compounds for anti-inflammatory and/or any immune supportive properties. The ability of the isolated compound to down-regulate pro-inflammatory cytokines, as was demonstrated here, can be of therapeutic importance. The crude extract suppressed IL-2, IL-4, IL-6 and IL-17A while the compound suppressed all cytokines tested except for IL-6 which was up-regulated. IL-6, which can act as a pro- or anti-inflammatory cytokine, is part of a network of cytokines that trigger or regulate immune responses and its increase by the compound in comparison to the inhibitory effect on pro-inflammatory cytokines contributes a beneficial balance to the host [25,45].

IL-2 and IL-17A have been reported to be responsible for activating pathogenic inflammation in a number of inflammatory skin diseases such as psoriasis [46,47]. High levels of IL-17A are reportedly associated with several chronic inflammatory diseases including rheumatoid arthritis and multiple sclerosis [48,49]. Therefore, lowering these cytokines in such cases will be of clinical use to the affected individual.

The down-regulation of the following cytokines IL-4, IL-10 and TNF by the extract and compound can be of general therapeutic significance. Over expression of these cytokines has been linked to certain autoimmune diseases as well as activation of pathogenic inflammation [46,47]. Inhibiting IL-4 has been associated with alleviating allergies while the neutralization of IL-10 has been linked to the reduction of helminth infection by restoring the function of Th2 effector cells [50].

The production of TNF was drastically inhibited by the labdane diterpenoid compound and this is a pro-inflammatory cytokine associated with cancers and autoimmune diseases [51]. TNF-α is regarded as a major pro-inflammatory cytokine and there is a continuous search for potential TNF-α inhibitors from natural products due to serious adverse side-effects of existing protein-based inhibitors [26]. Overproduction of TNF-α in rheumatoid arthritis patients has been linked to increased ROS [52]. Inhibiting high levels of this cytokine in such patients should also decrease ROS production and consequently inflammation and cellular damage. TNF-α has also been reported to induce NF-κβ production [53] and this protein is inhibited by the presence of antioxidants [54]. Here, *O. labiatum* showed antioxidant activity and the ability to lower the production of TNF-α meaning it could suppress NF-κβ production, further supporting this plant being potentially useful in diseases with pathogenesis enhanced by NF-κβ.

Different signalling pathways exist, including NF-kB, AP-1 and MAPK. Inhibition of each pathway can result in lower levels of specific cytokines such as IL-2 being produced due to inflammation [55]. The isolated labdane diterpenoid significantly inhibited c-Jun in the AP-1 pathway at 50 μM, indicating that inhibition of AP-1 is the mechanism by which the isolated compound exhibited its anti-inflammatory behaviour. The extract significantly stimulated c-Jun in AP-1 suggesting that the presence of other components in the extract could have resulted in a synergistic stimulatory effect on AP-1 production.

The reduction of INF-ɣ by the labdane diterpenoid in this study is suggestive of therapeutic potential. INF-ɣ is reportedly involved in the inflammatory events underlying a vascular inflammatory condition called abdominal aortic aneurysms (AAA) [56]. A large quantity of INF-ɣ was reported [56] in supernatants from AAA explant cultures that suggested this cytokine to be involved in AAA pathogenesis. Regulating the production of INF-ɣ in AAA patients by reducing its production could assist in alleviating that inflammatory condition. Another study [57] reported high production of INF-ɣ in the brain and periphery to be involved in activating certain metabolic pathways which leads to an inflammation cascade that results in aging and aging-associated medical psychiatric disorders. Suppressing INF-ɣ in such cases could be beneficial in avoiding/minimising psychiatric disorders associated with aging.

The anti-inflammatory properties of *O. labiatum* by pro-inflammatory cytokines inhibition were supported by the ability of the extract and labdane diterpenoid to reduce the production of NO release. NO, which is regarded as a potent inflammatory mediator, was inhibited at concentrations that were not toxic indicating that the NO actions of the extract and compound are not attributable to cytotoxicity. The data presented here confirms the ability of diterpenoids to also possess anti-inflammatory abilities through NO inhibition as previously reported [9].

Antioxidation and anti-inflammation properties of plants are routinely investigated and are considered to be among the primary health benefits of natural products. In addition, antioxidative agents prevent the formation of a number of diseases including inflammatory diseases. The anti-inflammatory properties of the *O. labiatum* extract as well as that of the isolated compound strongly suggest the immune enhancing properties of this plant.

## Conclusion

The *in vitro* data obtained from this study demonstrated for the first time that *O. labiatum* has potent anti-inflammatory and antioxidant activity. This work encourages further investigations of *O. labiatum*’s potential use as complimentary medicine in anti-inflammatory and antioxidant therapeutics and provides some empirical data for the already prominent anecdotal use in traditional medicines.

## Abbreviations

AP-1: Activator protein 1
CBA: Cytometric bead array
CUPRAC: Cupric reducing antioxidant capacity
DPPH: 2,2-diphenyl-1-picrylhydrazyl
FRAP: Ferric reducing antioxidant power
IL: Interleukin
MTS: 3-(4,5-dimethylthiazol-2-yl)-5-(3-carboxymethoxyphenyl)-2-(4-sulfophenyl)-2H-tetrazolium, inner salt
MTT: 3-(4,5-dimethylthiazol-2-yl)-2,5-diphenyltetrazolium bromide
NF-κB: Nuclear factor kappa B
NMR: Nuclear magnetic resonance
NO: Nitric oxide
PBMCs: Peripheral blood mononuclear cells
ROS: Reactive oxygen species
RT-CES: Real time cell electronic sensing
TNF: Tumour necrosis factor
